# How difficult is the validation of clinical biomarkers?

**DOI:** 10.12688/f1000research.6395.1

**Published:** 2015-04-28

**Authors:** Jan Voskuil

**Affiliations:** 1 Everest Biotech Ltd, Upper Heyford, OX255HD, UK

**Keywords:** biomarkers, antibodies, validation

## Abstract

Recent developments of introducing stratified medicine/personal health care have led to an increased demand for specific biomarkers. However, despite the myriads of biomarkers claimed to be fit for all sorts of diseases and applications, the scientific integrity of the claims and therefore their credibility is far from satisfactory. Biomarker databases are met with scepticism. The reasons for this lack of faith come from different directions: lack of integrity of the biospecimen and meta-analysis of data derived from biospecimen prepared in various ways cause incoherence and false indications. Although the trend for antibody-independent assays is on the rise, demand for consistent performance of antibodies (both in choice of antibody and how to apply it in the correct dilution where applicable) in immune assays remains unmet in too many cases. Quantitative assays suffer from a lack of world-wide accepted criteria when the immune assay is not ELISA-based. Finally, statistical analysis suffer from coherence both in the way software packages are being scrutinized for mistakes in the script and remaining invisible after small-scale analysis, and in the way appropriate queries are fed into the packages in search for output that is fit for the types of data put in. Wrong queries would lead to wrong statistical conclusions, for example when data from a cohort of patients with different backgrounds are being analysed, or when one seeks an answer from software that was not designed for such query.

## Introduction

Clinical biomarkers have been around for a long time now, and the field is moving rapidly. In addition to genetic and protein markers, we now also have microRNAs, epigenetic markers, lipids, metabolites, and imaging markers. Some are extremely useful as a (companion-) diagnostic; others may serve as a mere indicator. However, there are problems. There is confusion on the nomenclature and on the way how biomarkers are meant to be validated and used. A proposal published in 2006 was meant to create some clarity and consistency in the matter ^1^. The biggest obstacle by far is that Biomarker validation and qualification depend on confirmation at different locations (different labs). There are issues with consistency in the preparation of the biological material used in the different studies, and with consistency in the choice of antibody when required. It should also be noted that in quantitative immunohistochemistry (IHC) one needs a standard in the quantification method ^2^. A recent opinion paper reveals yet another layer of complexity: The statistical analysis is prone to wrong conclusions down to coding errors in the software ^3^. It may not be a surprise then that one another led to the observation that only about 11% of preclinical research papers demonstrated reproducible results ^4^. It is time to take stock and to address the different levels of disturbance complicating the process of biomarker validation and qualification.

## Biological material

The integrity of the tissue specimens will determine the quality of the biomarker’s measurements, especially when biomarkers are instable. Post-mortem samples in particular will never represent samples from living individuals because of the post-mortem delay. As the post-mortem delay will differ from individual to individual, the level of decay will vary dramatically per sample. For this reason, post-mortem samples are best fit for qualitative analysis. Quantification of any biomarker in post-mortem samples should be interpreted with extra care ^5^.

Plasma samples can be prepared in different ways: they can be prepared either by citrate, by ethylenediaminetetraacetic acid (EDTA) or by heparin. In addition, biomarkers can be tested in serum and in whole blood. It is clear that levels of biomarkers will need to be compared between equally treated samples in order to avoid variations in noise from the different ways the samples were prepared ^6^. Since this principle is universal, it will be true for any other tissue types.

For microscopy, tissue slides and cell suspensions have to be prepared in line with the required assay before they can be investigated. Fixatives (alcohols, aldehydes), embedding materials (paraffin, LR White, etc) and temperatures (frozen vs heated) have profound effects on the integrity of the tissues and cells and they will determine the success of the assay. Again, consistency in the tissue preparation, tissue sections and cells to be analysed is paramount ^7, 8^. Mega-data analysis may get skewed when data are collated from samples treated in different ways.

A systematic approach to record and keep biospecimen has been proposed and is aimed to become the new standard: Biospecimen Reporting for Improved Study Quality (BRISQ) guidelines provide a tool to improve consistency and to standardize information on the biological samples ^9^.

## Antibody choice

Mass-spec and RT-PCR quantifications will be robust by the consistency of the assay material. However, the robustness of immune assays depends highly on the choice of antibodies used in the assay. Once an antibody has been successfully validated in one assay, this assay is defined by this antibody. Change of antibody will potentially change the outcome altogether as demonstrated in the past ^10, 11^. When an antibody needs changing, the assay is no longer validated and the validation procedure will have to be repeated with the new antibody. For this reason the preference goes to monoclonal antibodies. The rationale behind this preference is that the clone number of the antibody would define its characteristics: the expectation then is that the assay will remain validated because the antibodies remain identical when using antibodies from the same clone number, no matter which vendor they are from. Unfortunately this is a myth. Depending on the vendor (and sometimes depending on the catalogue number) the formulations, all with the same clone number, will differ: the antibody may be purified from ascitic fluid, from culture media, or not purified at all (just ascitic fluid or just culture supernatant). These different formulations will have an effect on the way the antibody needs to be diluted to avoid non-specific background ^12^. Therefore, the monoclonal antibody needs to be revalidated in the same assay when the original formulation is no longer available. But even subsequent batches from the same formulation show some level of differences, thus undermining the main argument of preference to use monoclonal antibodies in standard assays. A peptide-generated polyclonal antibody from a larger animal than rabbit (for large size batches) may serve as a cost-effective alternative because the batch-to-batch variation of such antibody is limited by the size of the immunizing peptide unlike other polyclonal antibodies ^12^.

## Assay development

When a new assay is being developed a monoclonal antibody may not be always readily available. Then a peptide-generated polyclonal antibody may serve as a good and cost-effective alternative. However, peptide polyclonal antibodies need a new round of validation when a new batch from a different animal arrives, just like different formulated monoclonal antibodies.

During assay development it is essential to dilute the antibody far enough to avoid non-specific background, but it needs to be strong enough to allow measuring a dynamic range, especially when the assay is quantitative. When the assay is dependent on a secondary antibody, this antibody needs validation as well (with and without primary) so to assess its non-specific signals (noise) ^12^.

Specificity needs to be addressed by comparing specimen spiked and un-spiked with the intended protein of interest (analyte) at various quantities. The signals need to be proportionate to the spiked quantities. In addition, specimen known not to have any of the analyte needs to be compared with specimen known to have the analyte at natural levels ^13^.

## Detection and cut-off values

Sensitivity is commonly attributed to the antibody used in an assay, but this is a misunderstanding. Sensitivity is determined by the detection method of which the antibody/or primary and secondary antibodies may take part in. If levels of the analyte are low, a higher sensitivity is required. This increased sensitivity is usually not accomplished by increasing the antibody concentration, although using an antibody with higher affinity will help to some extent. But in general the change of detection method (fluorophore, isotope, PCR, etc.) is the appropriate step to take. Together with the increase of sensitivity, the noise and background will also increase. When a change to a higher sensitivity is required, the validation should focus on a more stringent regime for keeping noise and background at bay ^12^.

When quantification is a requirement, cut-off values need to be put in place. Both the Lowest Levels Of Quantification (LLOQ) and Highest Levels Of Quantification (HLOQ) must be determined. Often the detection limits are determined as well, but this is only relevant for qualitative work. In IHC these values become tricky, because the intensity of signal is not just a number generated by a detector; the density of signal is combined with the location in the tissue. In addition, the surface area of quantification needs well defined boundaries. And even when all these measures are in place, the quality of the tissue and the quality of the slides can potentially jeopardize these measures and skew the results ^14^. Diagnostics by IHC is therefore prone to misinterpretation when for one specific test consistency at all levels (same antibody at same dilution, identically prepared tissue samples, identical area surface, identical staining analysed, etc.) is not followed in all laboratories in the world.

## Statistics and jumping to conclusions

Statistical analysis is notoriously used to provide the convenient evidence required by the author(s). No matter what method of statistics is used, when the input data have been selected from a larger set, any outcome will be biased and flawed by default. Only analysis of ALL data (non-selected) would yield proper results, but then they might be inconclusive or inconvenient. The pressure to publish in peer-reviewed papers force authors to present statistics in the most incomprehensible way possible, knowing that their peers will not admit their confusion and likely take the author’s word for it ^15^. Even when the statistic results are sound, they may get over-interpreted. Thus original claims were made based on prejudice and weak statistics and only over time, when more scientific details become available, a more complex picture emerged. For example how cholesterol levels are linked to cardiovascular disease ^16, 17^, how cancer is not merely caused by mutations ^18, 19^, how obesity is not a choice of lifestyle ^20, 21^ etc. Simplified claims can be (and has been) driven by apparent conflicts of interest as suggested in a study ^22^. The reputation of biomarkers has suffered dramatically from lack of scientific integrity and as a result many scientists lost faith in the usefulness of biomarker databases. New guidelines have been introduced by publishers in order to introduce a new standard on how statistics are presented ^23^.

There are several statistical packages on the market for scientists and clinicians to use. However, these packages are quite advanced and need expertise handling, very much like a driver’s licence is required in order to safely use a motorised vehicle on the public road. Vendors of such packages admit that their products are not always properly used (personal communications). The chosen algorithms need to be appropriate for the type of data to be analysed: some algorithms are designed for decision making, and they are not necessarily fit for scientific fact finding. In addition, the same data entered in the same system may result in different output on different occasions simply because the wrong type of results is being asked for (personal communications with statistic analysts). Finally, subtle coding errors in the software cannot always be identified in small tests on script integrity, only to skew results when large scale data are being processed ^3^.

## Project design and personalized medical care/stratified approaches

When all the above hurdles have been successfully taken, we are not quite there yet. Each individual is different from the next, and therefore each individual has different tolerance or sensitivity to toxins and medicines. This makes the assessment of biomarkers to follow the progress of a disease, or to follow the efficacy of a therapy, difficult to analyse when a group of patients have been treated all in the same way but the individuals in the groups are so diverse in genetic and/or ethnic background that the data can still be all over the place. Only when a group is defined by a certain genetic or environmental background, would there be sufficient homogeny to assess a biomarker for this particular defined group. For example, only recently it was found that HER2-type breast cancer patients do not benefit as well from therapies when they carry PICK3CA mutations compared to those who do not ^24^. It is like the chicken-egg (catch-22) paradigm: one has to start clinical trials in order to identify the non-responsive patients and only then one can leave them out for proper validation of a new biomarker. However, proper validation demands positive and negative controls and not allowing to select the convenient data only. Although this paradox can be dealt with properly, it is no surprise that the search for proper clinical biomarkers remains very challenging for some time to come.
