# Supplementary material for: How difficult is the validation of clinical biomarkers?
Source: F1000Res. 2015 Apr 28;4:101. [Version 1] doi: 10.12688/f1000research.6395.1 (PMC4431379; doi:10.12688/f1000research.6395.1)
Supplement: Peer review history

# Peer review: Referee response for version 1

**DOI:** 10.5256/f1000research.6861.r8491

**Published:** 2015-05-11

**Authors:** Ijaz S Jamall

**Affiliations:** 1 Risk-Based Decisions, Inc., Sacramento, CA, USA

The paper “How difficult is the validation of clinical biomarkers?” by Jan Voskuil is timely in that as the author points out the burgeoning growth of biomarker assays particularly in chronic diseases and notably in cancer has led to the misinterpretation of both research and clinical data for all of the reasons pointed out by the author.

As correctly noted by the author, there are huge variations in sample collection, storage, preparation, assay used, antibodies involved, and analysis—all of which can lead to wide variability in results and confound the end user of such data. The author correctly points out steps that can be taken early on in the process that can minimize or preclude the accuracy of the results so obtained. There is a need for standardization. Furthermore, the statistical analyses of biomarker data need to be stipulated before the data are collected and not afterwards, using statistical software in a black-box manner to see what results might show statistical significance, even though the significance may be serendipitous or not clinically relevant ( Zapf *et al.*, 2015). The slavish adherence to standard software packages by many physicians and biomedical scientists would be amusing if the wider implications of their innumeracy were not so dire ( Partin *et al*., 2013; Tuppin *et al., *2012).

In order for a biomarker to be useful, it must reflect a change in concentration in the media sampled with a change in disease status. It is frequently assumed that serum or blood are the best media for the study of biomarkers but because of the number of potentially confounding variables in serum or blood, tears and saliva, because they reflect intracellular fluids, might serve as better indicators of intracellular events long before these are reflected in the blood ( Pieragostino *et al*. 2015; Salvisberg *et al*., 2014).

I recommend acceptance of this very interesting paper for indexation.

I have read this submission. I believe that I have an appropriate level of expertise to confirm that it is of an acceptable scientific standard.

# Peer review: Referee response for version 1

**DOI:** 10.5256/f1000research.6861.r8494

**Published:** 2015-05-06

**Authors:** Bjorn LDM Brücher

**Affiliations:** 1 Theodor-Billroth-Academy, Munich, Germany

Thank you for inviting me to review the paper from Jan Voskuil.

I enjoyed reading the manuscript “ *How difficult is the validation of clinical biomarkers*?” by Jan Voskuil and I agreed to review because of the following three aspects:

 

1. I appreciate the open review process of *F1000Research *and that such are published, as reviewers should stop hiding behind anonymity. There is – at least to me – no criterion justifying such if science increasingly wants transparency which we all know science needs.
2. The article provided is a must read. I would assume especially within the Biotech community but also scientists and clinicians should do so.
3. After I read the article the 2 ^nd^ time, I decided to write a review but not in the usual way of reviewing a manuscript for a journal, because the necessary aspects have already been included in a comprehensive manner. My intent was including comments as well as additional aspects which may be of importance from the aspect of a clinician, surgeon and scientist for helping to see the subject *biomarker* from additional and different aspects.

 

**General**

The author provides important aspects by critically evaluating the use of biomarkers. This is necessary as the author reminds us about reality and wishes in science as well as in clinical practice.The different headlines are well thought through and chosen critically while questioning issues surrounding standardization. This is even more important as reports published for biomarkers under investigation are not standardized and make the same mistakes which had been made in the 70s and 80s in terms of tumor markers. Despite the necessity for being critical, it should not be viewed as being a synonymous with negative behavior. The author takes the responsibility by addressing major obstacles and missing data and that is without doubt highly appreciated and needed.

Biomarkers in diagnosis and treatment of diseases are measured characteristics and reflect a biological state of a disease. In terms of cancer, there is hope that biomarkers will provide a detection and screening tool for diagnosis, treatment with an influence on outcome orientated patient stratification as well as on predicting and monitoring multimodal treatment. The ideal biomarker is objectively measured in a comprehensible way independent of which laboratory it is investigated in, is easily measurable, cost-effective, and evaluated as an indicator for pathological and/or biological processes and of consistent value across differences in age, gender or ethnicity. Therefore, there is a necessity to remind us to not repeat history by including nearly any protein as a biomarker.

**Where are we?**

Many biomarkers are already declared by many companies to determine or diagnose a disease, although no data of half-life, metabolism or different interaction by different pathways are known nor provided. Again, it seems that history repeats itself as we get into the same discussions and situations as during the 70s and 80s in regard to tumor markers and cancer. Therefore, it is of importance that the author attempts to structure this theme into the headlines biological material, antibody choice, assay development, detection of cut-off values, statistics and project design.

**Project Design**

Logistically I would have thought that having the sub-headline Project Design earlier in the paper as this would indicate the direction.

**Biological material**

Of course there is hope that biomarkers help detecting a disease or serving as a screening tool for making a diagnosis. So far, it is not clear which biological material should be used and also it is not clear if this changes occur during different stages of diseases. Further, there is no standardization on how which biological material is stored and which variables influence the quality of the assay. There is another underestimated variable which needs to be taken into account and using cancer as an example may reveal further problems:

Cancer is not one disease and contains a heterogeneous set of dysfunctions such that the information available for biomarkers are also heterogeneous. This gets worse if we remind ourselves about the following: Igarashi, et al., observed in 93 specimens investigated for  tumor microvessel density (MVD) and thymidine phosphorylase (dThdPase), that tumor cells strongly stained for TP were “…often observed as a rim in the periphery of the tumor nest” ^1^. On the other hand, biomarkers are not just expressed within tumor cell nests. Takebayashi, et al., revealed in 1998 that normal esophageal tissue showed a TP expression rate of 12.3% compared to 50.9% in the tumor cell area of 163 investigated resected ESCC, although the percentage rate of cells expressing TP was less than 5% in 85.9% of non-neoplastic tissues ^2^. Additionally even histomorphological tumor-negative lymph nodes (pN0) showed a TP expression rate of 27.9%. These examples illustrate that use of biomarkers needs a standardization in many aspects and by this scientists and clinicians need to be involved both trying to bring together the necessary aspects for future use of biomarkers, because the examples used could also mean that the gene expression is different in terms where a biopsy is taken as well as where apart of the specimen is cut for investigating biomarkers. Now, this may even be more complicated using fluids and under which condition they had been sampled, stored: was it during an operation? Do we know if drugs influenced those biomarkers of investigation with a short half-lives?

**Methods (antibody choice, ..)**

To my knowledge there is no standardization of methods in use for different biomarkers. Again, we had that during the 70s and 80s in terms of tumor markers in use and it took long to resolve. Is there a standard protocol in use for determination of microRNA? Is it not that some use RT-PCR, Northern blotting, oligo-based arrays, *in situ *hybridization, different assays (together with different samples)? Are these different techniques comparable?

**Statistics (detection of cut-off values, statistics, …)**

Cut-offs can be determined of course. How many papers do you know in which a group determines all available necessary variables, such as sensitivity, specificity, positive predictive value, negative predictive value and overall accuracy? The decision-making for a cut-off is ultimately a clinical decision, as the clinician determines what is most important to know. For example, if we do not want to overlook a patient then it can be assumed that it makes sense having a high sensitivity in terms of using response. How is this in terms using a ROC analysis (receiving operator curve analysis)? Using a ROC analysis for better determination of a threshold, we need to compare the different measurements of a biomarker against a gold standard. As addressed below, it gets even more complicated: if there is a standard in use, but we know that it is not justified declaring it as a gold standard, what should we do?

Furthermore,as the author points out clearly how correct observations during the past resulted in wrong conclusions, which even increased dogma behavior in cholesterol levels associated with cardiovascular disease, lifestyle and obesity and also in terms of vitamin intake and health, as well as the somatic mutation theory being the cause for cancer.

***An apple found in a car is not synonymous with the proof that apples grow in cars.***

           

Critical thinking and re-thinking is continuously needed for excellence in science as well as for useful approaches in daily clinical work. Someone may enjoy reading the recent critical remarks about the wear and tear of guidelines ^3^. I would argue that these views are also a must reading for the future implications of biomarkers.

**Clinical Response Classification**

This section is of course a huge one and has multiple aspects and due to this we cannot expect that all aspects are addressed by the author. However, this aspect is extremely important, as response is in daily clinical use and therefore I would assume, there is a must as a scientific and clinical reviewer addressing some points. One major question is: Can tumor response to therapy be predicted, thereby improving the selection of patients for cancer treatment?

This is a major problem now because if a biomarker is measured against the gold standard. But, is it justified declaring clinical response evaluation serving as gold-standard? The readers need to make up their own minds about the following which have recently been addressed as well ^4^. The response classification is in use since 1971 since the publication by Miller ^5^. What no-one wants to see is the fact, that this response classification is based on one experiment only which was conducted by Moertel and Hanley in 1976 ^6^.

Experiment

16 experienced oncologists (be aware that, in 1976, there was no definition of an oncologist and there wasn’t one of an expe­rienced oncologist either) in which they cov­ered solid wooden spheres with a layer of rubber foam and placed them on a soft mattress. The colleagues had to measure the diameter of these spheres in a random order using rulers or cali­pers. The analysis showed that there was an error of 25% in the measurement of the size of identical spheres in 25% of the measure­ments, and that an error of at least 50% occurred in 6.8% of the measurements.

This means that the clinical response classification in use was based on a single experiment, and is still in use since some 35 years. Only some minor modifications were done: the US National Cancer Institute, together with the European Association for Research and Treatment of Cancer, proposed ‘new response criteria’ for solid tumors; a replacement of 2D measurement with measurement of one dimen­sion was made ^7^. Tumor response was defined as a decrease in the largest tumor diameter by 30%, which would translate into a 50% decrease for a spherical lesion ^8^. However, no subsequent standardized of this recommendation was carried out, and 35 years after the primary experiment, no additional studies with a struc­tured logistical way of accurate objective assess­ment of treatment response have been conducted or proposed. This opens an important question: If a biomarker is measured and analyzed according to the clinical response classification above, will this reflect biology as needed?

**Immune Response**

Another important variable to address contains immunological biomarkers in terms of response: Immunologists might just declare a response if immune-competent cells have been decreased and, possibly, without clinical signs of improvement of patient condition, or decrease of tumor size. But is it appropriate to declare a decrease of an immune-competent cell as a biomarker of any utility?

**Disease stage**

The 5 year survival rates for non-metastasized localized esophageal carcinoma according to the American Cancer Society (ACS) in stage I (IA and IB) range between 71 and 57& while they drop in Stage IIA on 46% and range in Stage III (IIIA, IIIB, IIIC) between 20 and 9% ^9^. Do we know if different disease stages in cancer are associated with different metabolism of different biomarkers?

**Influence of paths which may influence quality of biomarker measurements**

Let us take an example growth factor-beta (TGF-beta). It is known that inhibitors of this pathway, especially TGF-β1, block the proliferation and trigger apoptosis in malignant as well as in benign tumor cells, but with increasing tumor growth development as TGF-β1 resistance to targeted therapy develops ^10^^-^^12^. So, does this mean, that we are not aware that the function of a biomarker quality is independent from the disease, its stage how strong or weak the biomarker under investigation influence different paths? Do different situations in which biomarkers are measured mean different necessary views and if so, how can we judge those?

Another example: Epidermal growth factor receptor (EGFR; ErbB-1; HER1 in humans) is the cell-surface receptor for members of the epidermal growth factor family (EGF-family) of extracellular protein ligands ^13^. EGFR plays a critical role in tumor progression by stimulating cell cycle progression, invasion, and metastasis ^14^. Response measured by the EGFR-antibody Cetuximab in anticancer-treated patients does not correlate with the observed degree of EGFR expression in tumor tissue in patients with metastatic colorectal cancer ^15^^,^^16^.

How many of the declarations of so-called breakthroughs in measuring a biomarker are justified? I have no doubt, that it is important to measure different biomarkers, but the marketing and promotional one should not be goal of scientists. This may be seen as an ethical aspect as well, but it is unfortunate but necessary as increasingly observations are reported and determined correctly within publications, but afterwards statements with journalists implicate having a breakthrough result, as nearly every scientific finding these days are declared as such, which from my perspective is very unfortunate. This inflationary marketing way should not be followed.

**Conclusion**

Taken the aspects reviewed above together, I repeat my statement: **the article by Jan Voskuil provided is a must read**. Many more aspects are necessary taking into account for future evaluation and standardization of biomarkers under investigation.

I have read this submission. I believe that I have an appropriate level of expertise to confirm that it is of an acceptable scientific standard.

## References

[1] IgarashiMDharDKKubotaHYamamotoAEl-AssalONagasueN: The prognostic significance of microvessel density and thymidine phosphorylase expression in squamous cell carcinoma of the esophagus. *Cancer*.1998;82(7) :1225-1232952901210.1002/(sici)1097-0142(19980401)82:7<1225::aid-cncr3>3.0.co;2-e

[2] TakebayashiYAkiyamaSNatsugoeSHokitaSNiwaKKitazonoMSumizawaTTaniAFurukawaTAikouT: The expression of multidrug resistance protein in human gastrointestinal tract carcinomas. *Cancer*.1998;82(4) :661-6669477097

[3] Sitges-SerraA: Clinical guidelines at stake. *J Epidemiol Community Health.*.2014;68(10) : 101906-90810.1136/jech-2014-20386224700580

[4] BrücherBLDMBilchikANissanAAvitalIStojadinovicA: Tumor response criteria: are they appropriate?. *journal*.2012;8(8) :903-9062289466310.2217/fon.12.78

[5] MillerABHoogstratenBStaquetMWinklerA: Reporting results of cancer treatment. *Cancer*.1981;47(1) :207-214745981110.1002/1097-0142(19810101)47:1<207::aid-cncr2820470134>3.0.co;2-6

[6] MoertelCGHanleyJA: The effect of measuring error on the results of therapeutic trials in advanced cancer. *Cancer*.1976;38(1) :388-39494753110.1002/1097-0142(197607)38:1<388::aid-cncr2820380156>3.0.co;2-a

[7] TherassePArbuckSGEisenhauerEAWandersJKaplanRSRubinsteinLVerweijJVan GlabbekeMvan OosteromATChristianMCGwytherSG: New guidelines to evaluate the response to treatment in solid tumors. European Organization for Research and Treatment of Cancer, National Cancer Institute of the United States, National Cancer Institute of Canada. *J Natl Cancer Inst*.2000;92(3) :205-2161065543710.1093/jnci/92.3.205

[8] JamesKEisenhauerEChristianMTerenzianiMVenaDMuldalATherasseP: Measuring response in solid tumors: unidimensional versus bidimensional measurement. *J Natl Cancer Inst*.1999;17(6) :523-5281008862210.1093/jnci/91.6.523

[9] The American Cancer Society: Survival rates for cancer of the esophagus by stage.Last Medical Review: 03/20/2014(Last Revised: 03/02/2015) : Reference source

[10] AkhurstRJDerynckR: TGF-beta signaling in cancer--a double-edged sword. *Trends Cell Biol*.2001;11(11) :S44-S511168444210.1016/s0962-8924(01)02130-4

[11] RobertsABWakefieldLM: The two faces of transforming growth factor beta in carcinogenesis. *Proc Natl Acad Sci U S A*.2003;100(15) : 1018621-862310.1073/pnas.163329110012861075PMC166359

[12] BensonRJ: Role of transforming growth factor beta in breast carcinogenesis. *Lancet Oncol.*.2004;5(4) :229-2391505095410.1016/S1470-2045(04)01426-3

[13] Herbst RS: Review of epidermal growth factor receptor biology.*Int J Radiat Oncol Biol Phys.*.2004;59(2 Suppl) :21-261514263110.1016/j.ijrobp.2003.11.041

[14] LuiVW1GrandisJR: EGFR-mediated cell cycle regulation. *Anticancer Res*.2002;22(1A) :1-1112017269

[15] CunninghamDHumbletYSienaSKhayatDBleibergHSantoroABetsDMueserMHarstrickAVerslypeCChauIVan CutsemE: Cetuximab monotherapy and cetuximab plus irinotecan in irinotecan-refractory metastatic colorectal cancer. *N Engl J Med.*.2004;351(4) :337-3451526931310.1056/NEJMoa033025

[16] SaltzLBMeropolNJLoehrerPJ SrNeedleMNKopitJMayerRJ: Phase II trial of cetuximab in patients with refractory colorectal cancer that expresses the epidermal growth factor receptor. *J Clin Oncol.*.2004;22(7) :1201-12081499323010.1200/JCO.2004.10.182
